# Concurrent hepatitis and anterior uveitis in an immunocompetent patient with secondary syphilis: A case report

**DOI:** 10.1097/MD.0000000000042878

**Published:** 2025-06-13

**Authors:** Hamed Kheir, Emad Elazab, Haytham Hassan, Mohamed Kassab, Sufyan Hajjat, Mohammed Makin, Abubakr Hamad, Nehad Jaser Ahmad

**Affiliations:** a Department of Internal Medicine, Al Kharj Military Industries Corporation Hospital, Al Kharj, Saudi Arabia; b Infectious Diseases Department, Al Kharj Military Industries Corporation Hospital, Al Kharj, Saudi Arabia; c Department of Clinical Pharmacy, College of Pharmacy, Prince Sattam bin Abdulaziz University, Al Kharj, Saudi Arabia.

**Keywords:** anterior uveitis, hepatitis, rash, syphilis

## Abstract

**Rationale:**

Syphilis, a sexually transmitted infection caused by *Treponema pallidum*, is primarily spread through sexual contact and can lead to severe systemic complications if untreated.

**Patient concerns:**

A 42-year-old immunocompetent male presented with a nonpruritic papulosquamous rash sparing the palms and soles, which progressed to anterior uveitis and hepatitis.

**Diagnoses:**

Diagnosis was confirmed by positive rapid plasma reagin and *T pallidum* hemagglutination assay, alongside histopathological evidence of granulomatous hepatitis on liver biopsy and slit-lamp findings consistent with anterior uveitis.

**Interventions:**

The patient was hospitalized and treated with intravenous aqueous penicillin G (3 million units every 4 hours for 14 days).

**Outcomes:**

To our knowledge, this represents the first reported case in Saudi Arabia of secondary syphilis manifesting concurrently with anterior uveitis and hepatitis in an immunocompetent host.

**Lessons:**

The study underscores the importance of considering syphilis in atypical presentations to ensure timely diagnosis and management.

## 1. Introduction

Treponema pallidum is the causative agent of syphilis, a systemic bacterial infection transmitted exclusively among humans through sexual contact (sexually transmitted disease). The incubation period ranges from 10 to 90 days, with duration inversely proportional to the inoculum size.^[1,2]^ Untreated syphilis progresses through 4 distinct stages: primary, secondary, latent, and tertiary. Although approximately 30% of untreated patients exhibit cerebrospinal fluid (CSF) abnormalities indicative of neurosyphilis, most remain asymptomatic neurologically.^[1,2]^ Ocular manifestations may arise at any disease stage and can involve any ocular structure. Consequently, ocular syphilis should be considered in the differential diagnosis of nearly all infectious or inflammatory eye conditions.^[3]^ Despite its clinical significance, ocular syphilis remains underrecognized and is more prevalent in individuals coinfected with human immunodeficiency virus (HIV).^[4]^

Although recognized for decades, syphilitic hepatitis remains a rare and likely underdiagnosed manifestation of Treponema pallidum infection. Given the potential for severe morbidity if undetected, it should be considered in the differential diagnosis of patients with unexplained liver enzyme elevations.^[5]^ Clinically significant hepatitis can occur at any stage of syphilis, with an estimated 3% of secondary syphilis cases progressing to hepatitis. Fulminant hepatitis and cirrhosis are uncommon complications.^[6]^ Serological testing remains the cornerstone of laboratory diagnosis, providing provisional confirmation and enabling screening, diagnosis, and treatment monitoring. These tests are categorized into non-treponemal (e.g., rapid plasma reagin, VDRL) and treponemal (e.g., FTA-ABS, TP-PA) assays.^[7]^

## 2. Case presentation

**Figure 1.**
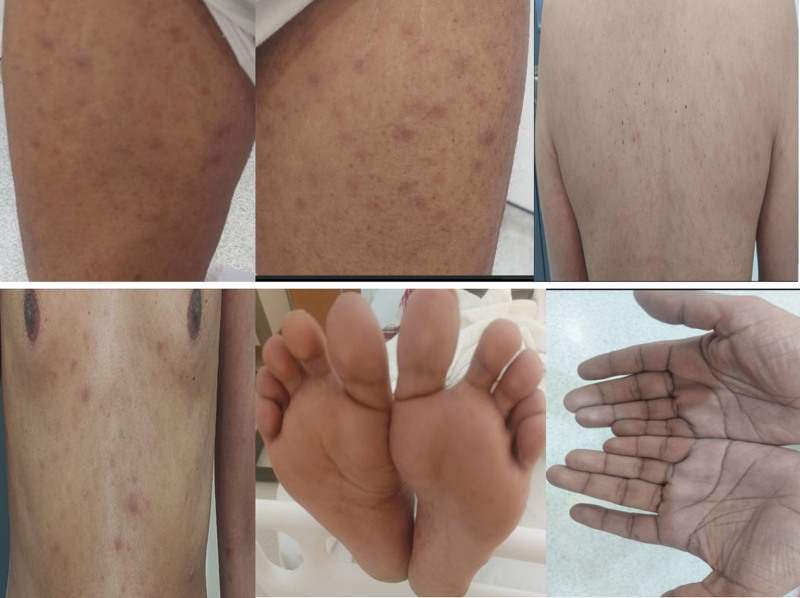
The development of maculopapular rashes. Maculopapular rash is a characteristic rash seen in secondary syphilis. It appears as red or brownish spots (macules) and raised areas (papules) on the skin. The patient exhibits maculopapular rashes that are widespread across the body, with the exception of the palms and soles.

A 42-year-old male presented to the emergency department with a chief complaint of generalized skin rash. Initially diagnosed with allergic dermatitis, he showed no improvement with symptomatic treatment. Subsequently, he developed blurred vision and ocular pain, prompting reevaluation. Ophthalmologic examination revealed anterior uveitis, leading to a diagnostic workup that included syphilis serology. Screening with rapid plasma reagin was positive, and the diagnosis was confirmed by a reactive Treponema pallidum hemagglutination assay. Upon infectious disease consultation, a diffuse maculopapular rash was noted, sparing the palms and soles (Fig. 1). The patient denied genital ulcers but reported a prior painless lower lip ulcer that was resolved before the rash appeared.

Review of the patient’s serial laboratory results revealed persistently elevated liver enzymes (ALT, ALP) and CRP across multiple emergency department visits, with ALT showing gradual but incomplete improvement. Initial infectious workup returned negative for HIV and viral hepatitis (HAV IgM, HCV Ab, and HBsAg). While CMV IgG was positive, PCR demonstrated a nonsignificant viral load (47 IU/mL). Additional investigations – including ASMA, ANA, alpha-1 antitrypsin, and ceruloplasmin – were unremarkable. Abdominal ultrasound showed no hepatosplenic abnormalities. The patient declined lumbar puncture. A diagnosis of secondary syphilis with neurosyphilis manifestations and syphilitic hepatitis was established. Treatment was initiated with intravenous aqueous penicillin G (3 million units every 4 hours for 14 days), supplemented with topical prednisolone eye drops. Within 48 hours, the patient exhibited marked clinical improvement: resolution of ocular pain, restored visual acuity, normalization of liver enzymes, and progressive rash fading. Notably, penicillin therapy resolved the previously unexplained hepatitic profile. ALT declined from 81 U/L in September to 39 U/L in October (reference: 4–36 U/L). Furthermore, ALP dropped from 1178 U/L in September to 595 U/L in October (reference: 44–147 U/L).

## 3. Discussion

Syphilis, caused by the spirochete Treponema pallidum, can lead to ocular involvement at any disease stage, affecting both immunocompetent and immunocompromised individuals.^[8]^ However, HIV coinfection significantly increases the risk, with syphilis patients living with HIV being nearly twice as likely to develop ocular symptoms compared to HIV-negative individuals.^[9]^ While ocular syphilis is more frequently reported in HIV-infected patients, rare cases in HIV-uninfected persons have also been documented. Although ocular syphilis is commonly linked to HIV coinfection, it can also occur in immunocompetent individuals, as demonstrated by our patient and previous case reports.^[10,11]^ For instance, McKibbin et al described a healthy 47-year-old man with ocular syphilis, while Roy et al reported a similar case in a 52-year-old immunocompetent patient.^[10,11]^ The increasing incidence of ocular syphilis in immunocompetent individuals suggests a need for heightened clinical suspicion, especially in high-risk populations. Risk factors such as delayed diagnosis, high-risk sexual behaviors, and strain-specific neurotropism may contribute. Enhanced screening, prompt treatment, and public health interventions targeting syphilis prevention are crucial to mitigating this trend.

The current case demonstrates an atypical presentation of secondary syphilis, characterized by a non-pruritic papulosquamous rash sparing the palms and soles, progressing to anterior uveitis and hepatitis. While Lleó et al describe the classic secondary syphilis rash as a widespread, copper-colored maculopapular eruption involving palmar and plantar regions, our case highlights the disease’s variable dermatologic manifestations, which may include nodular, pustular, annular, or frambesiform lesions in atypical presentations.^[12]^ Hepatic involvement, though uncommon, was a prominent feature in this case. Rubio-Tapia et al note that approximately 10% of syphilis patients exhibit elevated hepatic enzymes without other features of hepatitis, while clinically significant syphilitic hepatitis develops in about 3% of secondary syphilis cases.^[6]^ Our patient’s presentation aligns with these observations, demonstrating how syphilitic hepatitis can occur at any disease stage. The ocular manifestations in this case warrant special attention. Ocular syphilis can arise at any stage of syphilis infection and presents with diverse clinical features, ranging from isolated eye involvement to cases with concurrent neurologic symptoms. While it can affect nearly any part of the eye, posterior uveitis and panuveitis are the most frequent presentations, though anterior uveitis, optic neuropathy, retinal vasculitis, and interstitial keratitis may also occur. If untreated, it can cause progressive vision loss and permanent blindness. Notably, many patients with ocular syphilis lack systemic symptoms, particularly in secondary syphilis, where eye involvement may emerge up to 6 months after initial infection – often after other signs have resolved. However, in tertiary syphilis, roughly half of patients with ocular disease also exhibit systemic manifestations.^[13,14]^ Tuddenham and Ghanem suggest that in patients with isolated ocular symptoms and positive syphilis serology, CSF analysis may be unnecessary, as 40% to 90% of such cases show no CSF abnormalities.^[15]^ This supports our decision to proceed with treatment despite the patient’s refusal of lumbar puncture. Intravenous aqueous penicillin G (3 million units every 4 hours for 14 days) was administered as first-line therapy. While penicillin remains the gold standard, doxycycline serves as an effective alternative for early and late latent syphilis.^[16]^ The rapid resolution of symptoms and normalization of liver enzymes following penicillin therapy in our case underscores the importance of timely diagnosis and appropriate antimicrobial treatment.

## 4. Conclusion

While secondary syphilis classically presents with non-pruritic papulosquamous eruptions involving the palms and soles, their absence does not exclude the diagnosis. This case underscores the importance of considering syphilis in sexually active patients presenting with either unexplained hepatic enzyme elevations or uveitis. Notably, all patients with ocular syphilis should be managed as potential neurosyphilis cases, regardless of lumbar puncture results, given the significant risk of neurological involvement.

## Acknowledgments

This study is supported via funding from Prince Sattam bin Abdulaziz University project number (PSAU/2024/R/1446). We would like also to thank the patient and his family.

## Author contributions

**Conceptualization:** Mohammed Makin, Abubakr Hamad.

**Data curation:** Haytham Hassan, Mohamed Kassab.

**Methodology:** Sufyan Hajjat.

**Supervision:** Hamed Kheir.

**Writing – original draft:** Nehad Jaser Ahmad.

**Writing – review & editing:** Emad Elazab.
